# Balanced Hydroxyethylstarch (HES 130/0.4) Impairs Kidney Function In-Vivo without Inflammation

**DOI:** 10.1371/journal.pone.0137247

**Published:** 2015-09-04

**Authors:** Martin Alexander Schick, Wolfgang Baar, Raphael Romano Bruno, Jakob Wollborn, Christopher Held, Reinhard Schneider, Sven Flemming, Nicolas Schlegel, Norbert Roewer, Winfried Neuhaus, Christian Wunder

**Affiliations:** 1 Institute of Anaesthesia and Critical Care, University Hospital Würzburg, Würzburg, Germany; 2 Department of Anesthesiology and Intensive Care Medicine, University Medical Center Freiburg, Freiburg, Germany; 3 Department of Medicinal Chemistry, University of Vienna, Vienna, Austria; 4 Department of Internal Medicine I, University Hospital Würzburg, Würzburg, Germany; 5 Department of general, visceral, vascular and paediatric surgery (Department of Surgery I), University of Würzburg, Würzburg, Germany; 6 Institute of Medical Genetics, Medical University of Vienna, Vienna, Austria; Robert Bosch Hospital, GERMANY

## Abstract

Volume therapy is a standard procedure in daily perioperative care, and there is an ongoing discussion about the benefits of colloid resuscitation with hydroxyethylstarch (HES). In sepsis HES should be avoided due to a higher risk for acute kidney injury (AKI). Results of the usage of HES in patients without sepsis are controversial. Therefore we conducted an animal study to evaluate the impact of 6% HES 130/0.4 on kidney integrity with sepsis or under healthy conditions Sepsis was induced by standardized Colon Ascendens Stent Peritonitis (sCASP). sCASP-group as well as control group (C) remained untreated for 24 h. After 18 h sCASP+HES group (sCASP+VOL) and control+HES (C+VOL) received 50 ml/KG balanced 6% HES (VOL) 130/0.4 over 6h. After 24h kidney function was measured via Inulin- and PAH-Clearance in re-anesthetized rats, and serum urea, creatinine (crea), cystatin C and Neutrophil gelatinase-associated lipocalin (NGAL) as well as histopathology were analysed. In vitro human proximal tubule cells (PTC) were cultured +/- lipopolysaccharid (LPS) and with 0.1–4.0% VOL. Cell viability was measured with XTT-, cell toxicity with LDH-test. sCASP induced severe septic AKI demonstrated divergent results regarding renal function by clearance or creatinine measure focusing on VOL. Soleley HES (C+VOL) deteriorated renal function without sCASP. Histopathology revealed significantly derangements in all HES groups compared to control. In vitro LPS did not worsen the HES induced reduction of cell viability in PTC cells. For the first time, we demonstrated, that application of 50 ml/KG 6% HES 130/0.4 over 6 hours induced AKI without inflammation in vivo. Severity of sCASP induced septic AKI might be no longer susceptible to the way of volume expansion.

## Introduction

Infusion therapy is a cornerstone in intensive and perioperative care to increase intravascular volume and to preserve macro- and microcirculation. In 2008, the VISEP-study results showed an increased risk of acute kidney injury (AKI) when 10% HES 200/0.4 solution was used in septic patients[1]. With 6S-, CRYSTMAS- and CHEST-study further trials demonstrated an increased incidence of AKI, when modern 6% HES 130/0.4 or 0.42 were infused[2–4]. Therefore the European Medicines Agency´s Pharmacovigilance Risk Assessment Committee (PRAC) recommended not to use hydroxyethylstarch in sepsis (EMA / 640658 / 2013), despite of the results of the CRISTAL-study. This study also reveals no differences in AKI using colloids (HES, gelatine, dextran or albumin) compared to crystalloids alone[5].

Large amounts of HES are still commonly used in perioperative care. The administration of starch is based on the widespread belief that this substance improves the treatment for these patients and therefore reduces morbidity. The pathophysiology of HES-induced AKI is not fully understood so far. Recently we demonstrated that HES induces decreased cell viability in human proximal tubules cells (PTC) in vitro (Bruno et al. A&A 2014)[6]. In this trial TNF-α did not worsen the harmful impact of HES on PTC, but only applied mass of HES molecules seemed to be responsible for the derogation of human PTC. All investigated HES solutions (potato or corn derived; balanced or non balanced) and molecular sizes (3–200 kDa) revealed PTC impairments. Therefore we conducted this study to evaluate, whether inflammation is the key trigger for HES induced AKI in vivo, or whether HES impairs kidney function even under healthy conditions in our previously published new rat model of septic AKI [7].

## Materials and Methods

### Animals

This study was carried out in strict accordance with the recommendation in the Guide for the Care and Use of Laboratory Animals of the National Institutes of Health. After animal care committee approval (PM-No. 83/11, Laboratory Animal Care and Use Committee of the District of Unterfranken, Germany), experiments were performed on 24 male Sprague-Dawley rats (324±16g bodyweight (BW)) purchased from Harlan Winkelmann (Borchen, Germany). Animals were treated according to the guidelines of the U.S. National Institutes of Health, as well as those of Germany. All rats were maintained on a standard diet and water ad libitum and 12 h day and night cycles. Animals were not fasted prior and after the surgical interventions.

**Fig 1:**
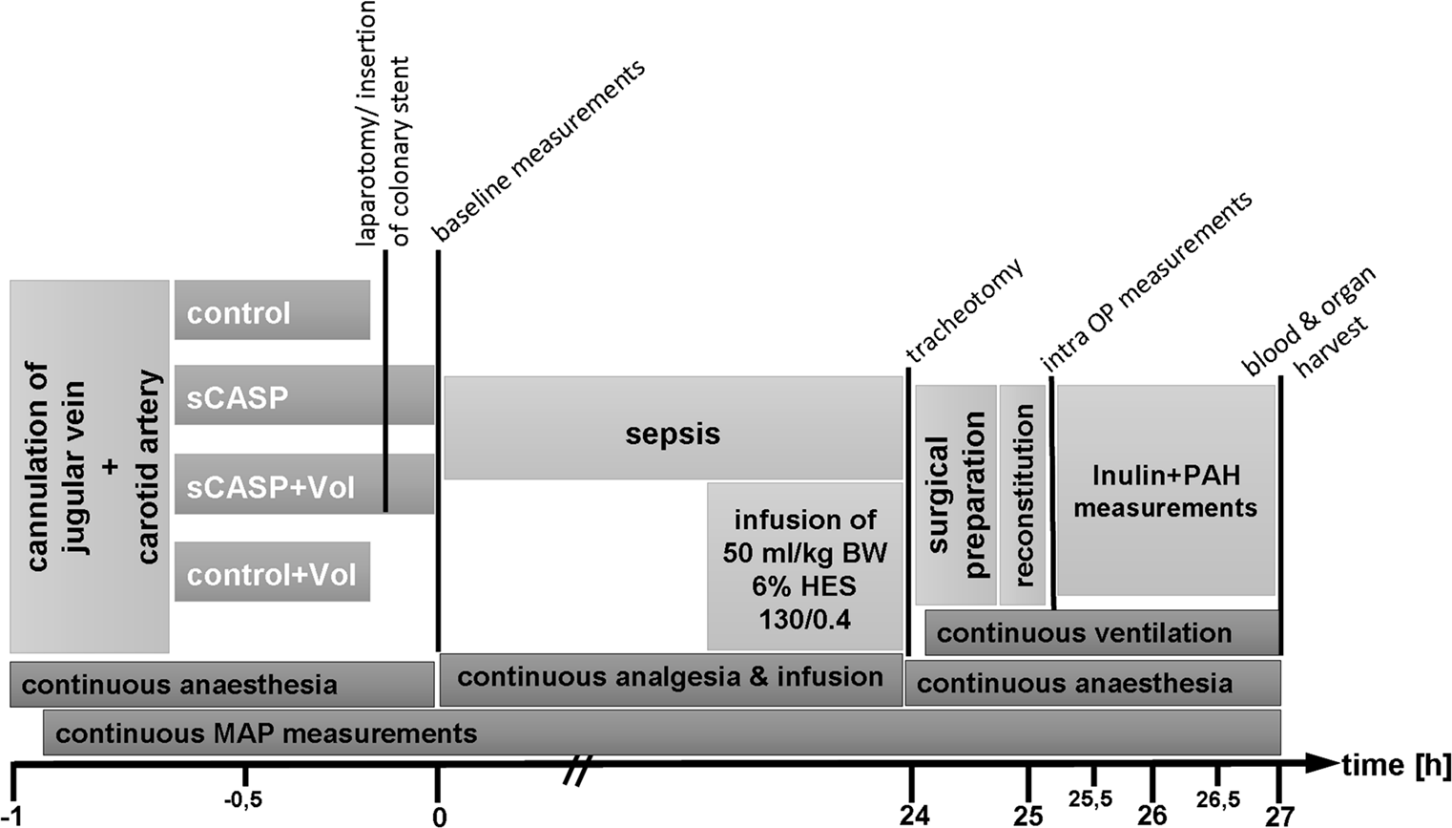
Schematic presentation of the experimental setup. At point of time 0 h after cannulation of the right jugular vein and the left carotid artery, sham or sCASP procedure was performed. Baseline measurements of macrohemodynamic and blood gas analysis followed after operation. 18 hours after operation, sCASP+VOL and Control+VOL received 50 ml/kg/BW 6% HES 130/0.4 (VOL) within the following 6 hours. Twenty-four hours after first operation, macrohaemodynamics and blood gas parameters were measured, and renal function was analysed by inulin- and PAH- clearance. At the end of experiment blood serum, blood gas analysis and kidney were harvested.

Animals were randomized to groups I–IV (n = 6/group, Fig 1), group I: Control, group II: Control+Vol [C+VOL] (balanced 6%HES 130/0.4, Volulyte, Fresenius Kabi, Germany), group III: sCASP, group IV: sCASP+Vol (balanced 6%HES 130/0.4, Volulyte, Fresenius Kabi, Germany), and anesthetized using isoflurane (Forene, Abbott, Germany) and nitrous oxide inhalation as described previously [7–9]. Briefly the right jugular vein was cannulated for infusion and medication purposes, and for continuous blood pressure measurement, heart rate (Hewlett Packard Model 88S) and gaining blood samples, the left carotid artery was also cannulated[9].

#### sCASP procedure

Animals of group III and IV received the sCASP procedure (for details see Schick MA et al. 2014 [7]). Spontaneously breathing anesthetized rats were placed in supine position on a heating pad to ensure normothermia (rectal probe 38±5°C). After thorough disinfection of the abdominal skin, a midline incision was performed. The peritoneal cavern was opened by incising the abdominal muscles and the peritoneum along the linea alba. The cecal pole was identified and the cecum was gently pulled out by the use of cotton swabs. 20mm distal from the ileo-cecal valve, the colon was punctured with a 14G needle and to ensure a continuous fecal peritonitis in male rats, a 10 FR plastic tube (tip of a suction catheter, type “Ideal”, B.BraunMelsungen, Germany) was fixed with a suture. Stool was milked from the cecum towards the colonic stent by the use of cotton swabs until it appeared at the outlet of the stent. Then the gut was replaced into the peritoneal cavity and the stent was flushed with 2 ml NaCL 0.9% (Fresenius Kabi, Germany) to distribute the faeces into the peritoneal cavern. Afterwards the peritoneum and the skin were closed with continuous two layer sutures.

After the surgical procedures, animals woke up and received the following treatments: Every animal received 14.4 ml/kgBW/24h NaCl 0.9% for basal fluid requirement and after 18 hours C+VOL and sCASP+VOL treated animals received 50 ml/kgBW balanced 6% HES 130/0.4 continuously over the following 6h additionally. All animals had free access to water and food. Analgesia protocol was performed as follows: Control and C+VOL received 0.25 μg/100g BW/h fentanyl (Fagron, Germany); CASP and CASP+VOL 2.0 μg/100g BW/h fentanyl intravenously. Invasive MAP was continuously monitored, and animals would have been excluded to further investigation if MAP would have dropped below 60 mmHg during the next 24h. However, all animals showed MAP measurements above 60 mmHg for the following 24 hours.

After 24 hours rats were re-anesthetized using Midazolam (Midazolam-ratiopharm, Ratiopharm, Germany) 0.7 mg/100g BW/h and Fentanyl 7 μg/100g BW/h. For a sufficient anesthetic depth, Isofluran was adapted afterwards[10]. Afterwards the animals were weighed again (322±22g). To establish standardised ventilation, a tracheotomy was performed and rats were mechanically ventilated with FiO_2_ 0.28% using a Rodent Ventilator (Type: 7025, Hugo Sachs Elektronic KG, Germany). For cardiac output (CO) measurements the right femoral artery was cannulated, and a thermocatheter (MLT1402 T-type Ultra Fast Thermocouple, ADInstruments) was introduced to measure CO as previously described [11]. Blood gas values (BGA) were measured 30 min after the surgical procedure on day one and on day two, 15 min after the tracheotomy, using ABL505 blood gas analyzer (Radiometer, Copenhagen).

#### Evaluation of kidney function in vivo

Inulin and PAH clearances were determined as described recently[12]. In brief, after BGA determination Fluorescein-isothiocyanate-inulin (Inulin-FITC; F3272–1; Sigma-Aldrich; St. Louis, USA) and PAH (p-Aminohippuric acid sodium salt; A3759–25G; Sigma-Aldrich; St. Louis, USA) (1 mg of each substance solved in 0.25 ml 0.9% NaCl) were applied (bolus 75μl i.v.), followed by constant infusion of both substances (2 mg/ml inulin, 5 mg/ml PAH) with a rate of 3.7μl/h/300gBW. The urine bladder was cannulated with a PE-50 (polyethylene) catheter to measure urine flow and obtain urine samples. After 30 min of infusion, a steady state was reached. Then urine was collected over a period of 20 min and blood samples were drawn subsequently. Samples were centrifuged and stored at −20°C. Inulin concentrations in urine and plasma were determined by fluorescence spectrometry (1420 Victor^2^Multilabel Counter), whereas PAH concentrations were measured by photo spectrometry (Dynatech Lab, Guernsey, UK) using the anthrone method. Calculations of inulin clearance, PAH clearance, and PNS were performed according to the equations: inulin clearance = (I_U_ × V_U_)/(I_P_ × t); PAH clearance = (PAH_U_ × V_U_)/(PAH_P_ × t); and PNS = [(PAH_U_ × V_U_)/t] − [GFR × PAH_P_]; where I_U_ is inulin concentration in urine; PAH_U_ is PAH concentration in urine; I_P_ is inulin concentration in plasma; PAH_P_ is PAH concentration in plasma; V_U_ is urine volume; PNS is PAH net secretion and t is time of measurement.

#### Markers of acute kidney injury

At the end of experiment plasma samples were drawn for determination of kidney function and damage by measuring creatinine and urea levels by using routine laboratory methods. For cystatin C and NGAL, blood samples were collected, prepared and measured as previously described using rat NGAL (Kit 041 dianova, Germany) and cystatin C (cystatin c ELISA KIT, AXXORA, Germany) kits[8].

#### Histopathology

Kidneys were harvested at the end of the experiment for histopathological studies. Organ tissues were fixed in formaldehyde 3.5% (Otto Fischar, Germany) for more than 24 h. Tissues were stained with haematoxylin and eosin reagent and PAS-reaction as previously described [8, 13]. The morphological alterations of kidneys were analysed semi-quantitatively by a blinded investigator where 0 was given when no alterations were found, 1 for mild alterations, 2 for medium alterations and 3 for severe alterations. Criteria for histopathological assessment were formation of oedema, cellular oedema, detachment of tubular epithelium from the basement membrane, loss of the brush border of the proximal tubular cells, cell death and vacuolisation. Mean values from the scores of the latter criteria were taken together as total injury score. Additionally, 10 randomly chosen glomeruli from tissue sections of each group were selected and distal and proximal tubular cells around the glomeruli were analysed under high-power fields (6300 magnification) and the score 0 to 4 was given: 0 = none; 1 = < 25%, 2 = 25–50%, 3 = 50–75% and 4 = > 75% of the epithelial cells displayed vesicles.

### In vitro

For the investigation of the impact of HES in vitro we used our established cell culture of human proximal tubule cells (HK-2) [6, 14]. In brief HK-2 cells were purchased from ATCC and used between passage 10 and 29. Cells were cultured in DMEM/HämF12 medium with 10% FCS and 1% penicillin/streptomycin (GIBCO, Invitrogen) for the EZ4U test and without pyruvat for the LDH tests. For cell viability and cytotoxicity assays 50000 HK-2 cells/cm^2^ were seeded on gelatine-coated 96-well plates. Different concentrations of HES were applied onto HK-2 cells. Cell viability was determined by means of EZ4U assay (second generation tetrazolium dye XTT, Biomedica) after 21 hours substance application according to the manufacturer’s instruction. For cytotoxicity (cyt) the Cytotoxicity Detection KIT (LDH-releasing, Roche Applied Science Product number 11644793001, Roche Diagnostics GmbH, Mannheim, Germany) was used after 24h according to the manufacturer´s instruction. This assay determines LDH activity released from damaged cells using the specific absorption of formazan dye measured with ELISA. Cyt was converted in percent using following equation: cyt = (measured value-low control)/(high control-low control)x100. High control equals the mean value obtained from wells with lysed HK-2 cells, while the low control equals to the mean measured value of HK-2 cells, treated with control solution. Balanced 6% HES130/0.4 solution was diluted in HK-2 medium to 0.1, 0.5, 1, 1.5, 2, 4% HES using for every dilution the same amount of HK-2 medium (27.5%). Residual volumes were filled up with NaCl solution (NaCl 0.9, Fresenius Kabi Germany). For estimating the influence of pro-inflammatory stimulation, cells were incubated with different concentrations of lipopolysaccharide (10–100ng/ml LPS from Escherichia coli 055:B5, purchased from Sigma, Germany). Results were related to control values from cells treated without HES130/0.4 (= 0% values with HK-2 medium and NaCl only). 0%-control values were set to 100% viability for graphical illustration.

### Statistics

Values throughout are expressed as mean ± SEM or SD. In vivo: Statistical analyses were performed using SPSS V. 20 and SPSS V. 21. For parametric parameters possible differences were assessed with ANOVA followed by post-hoc Duncan test. Statistical significance is assumed for p < 0.05. For non-parametric data Kruskal-Wallis following Mann-Whitney-U test with Bonferroni correction were used for significant differences. In vitro: For statistical analysis a two-sided t-test with different variances was used, p-values<0.05 and 0.001 were classified as statistical significant.

## Results

### In vitro

**Fig 2:**
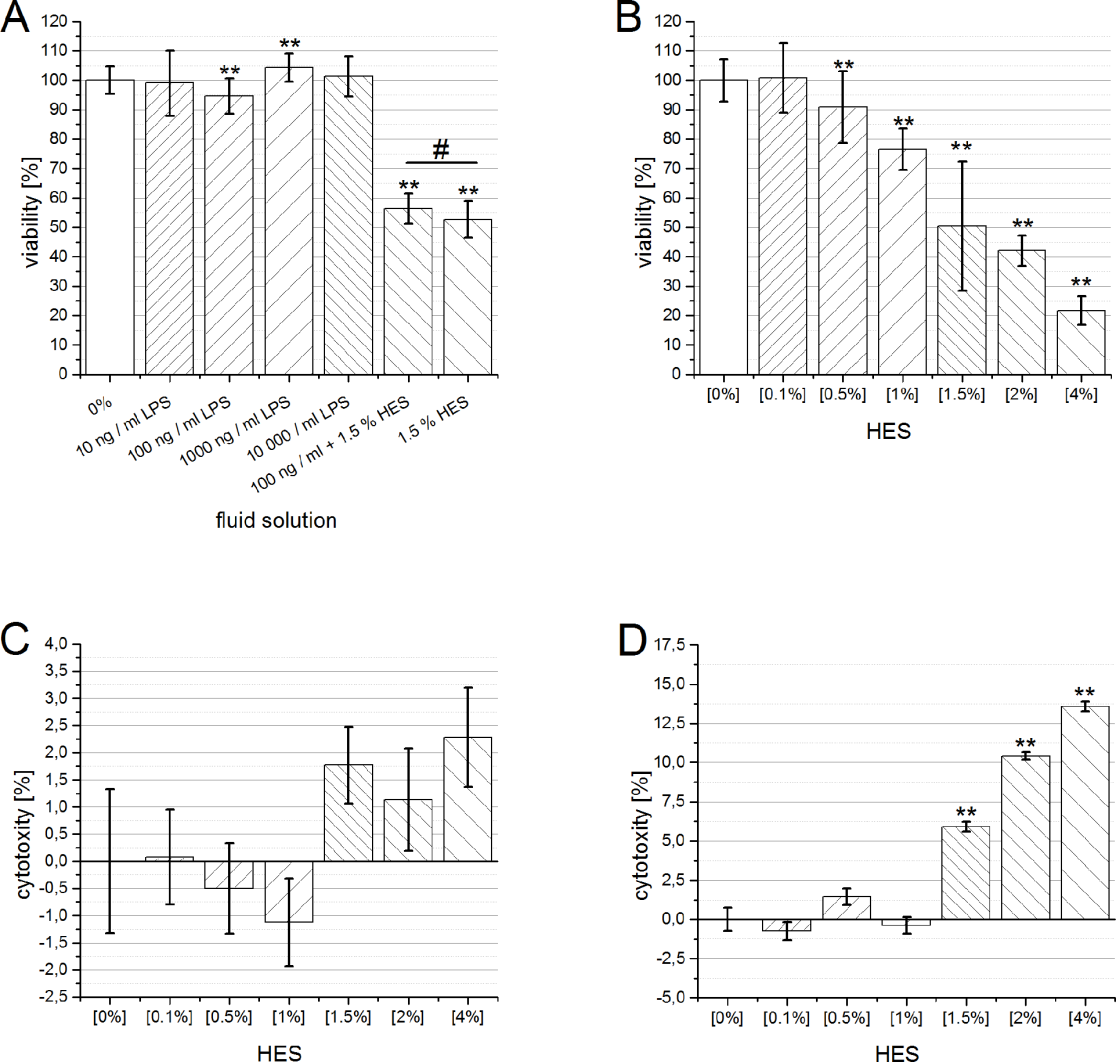
The influence of inflammation, incubation time on the viability and cytotoxicity of human proximal tubule (HK-2) cells. A: Relative reduction of cell viability by increasing doses of LPS and coincubation of LPS (100 ng /mL) plus 1.5% HES 130/0.4 and 1.5% HES alone, measured after 21 hours. Data are presented as mean ± SD (n = 36–48), **P < 0.001 versus control, # P < 0.01 vs. each other. B: Relative reduction of cell viability of HK-2 cells by increasing doses of HES 130/0.4, after 0 hour incubation. Data are presented as mean ± SD (n = 36–48), * vs. control: *P < 0.01, **P < 0.001. C/D: Relative increase of cytotoxity of HK-2 cells by increasing doses of HES 130/0.4, after 4 (C) and 24 h incubation. Data are presented as mean ± SD (n = 36–48). * vs. control: **P < 0.001, 2-sided *t* test with same variances.

In our previous studies, HES showed adverse effects on the kidney in sepsis. Therefore we assessed whether the primarily demonstrated harmful effect of HES is caused by inflammation. As previously shown for TNF-α [15], we investigated the impact of different dosages of LPS on cell viability (Fig 2), quantified with the XTT assay. Neither incubation with LPS caused a relevant derangement of cell viability, nor there was a relevant difference between 1.5% HES (52.66% ± 6.28% SD) and 1.5% HES co-incubated with 100 ng/mLLPS (56.46% ± 5.07%). Fig 2 shows the impact of HES on cell viability after 0h of incubation. The harmful effect of HES can be determined directly after the administration in vitro. Furthermore, the degree of damage is very similar to values measured in the later time course [6, 14]. Direct cell toxicity follows belatedly this direct decrease of cell viability. While after 4h no relevant increase of cell toxicity incubating with HES was detectable (Fig 2), the values observed after 24h of incubation reveal a dose depended (beginning with 1.5% HES) statistically significant cell damage (Fig 2: 1.5% HES: 5,92% ± 5,76% SD, 2% HES: 10,42% ± 5,64% SD and 4% HES: 13,57% ± 8,46% SD).

### In vivo—Determination of sepsis induced Acute Kidney Injury

Macrohemodynamics, arterial blood gas parameters and additional plasma analysis are shown in supplemental materials (S1 Table, S2 Table, S3 Table). All animals treated with sCASP displayed clinical features of sepsis, as described previously [7].

#### Functional Test

**Fig 3:**
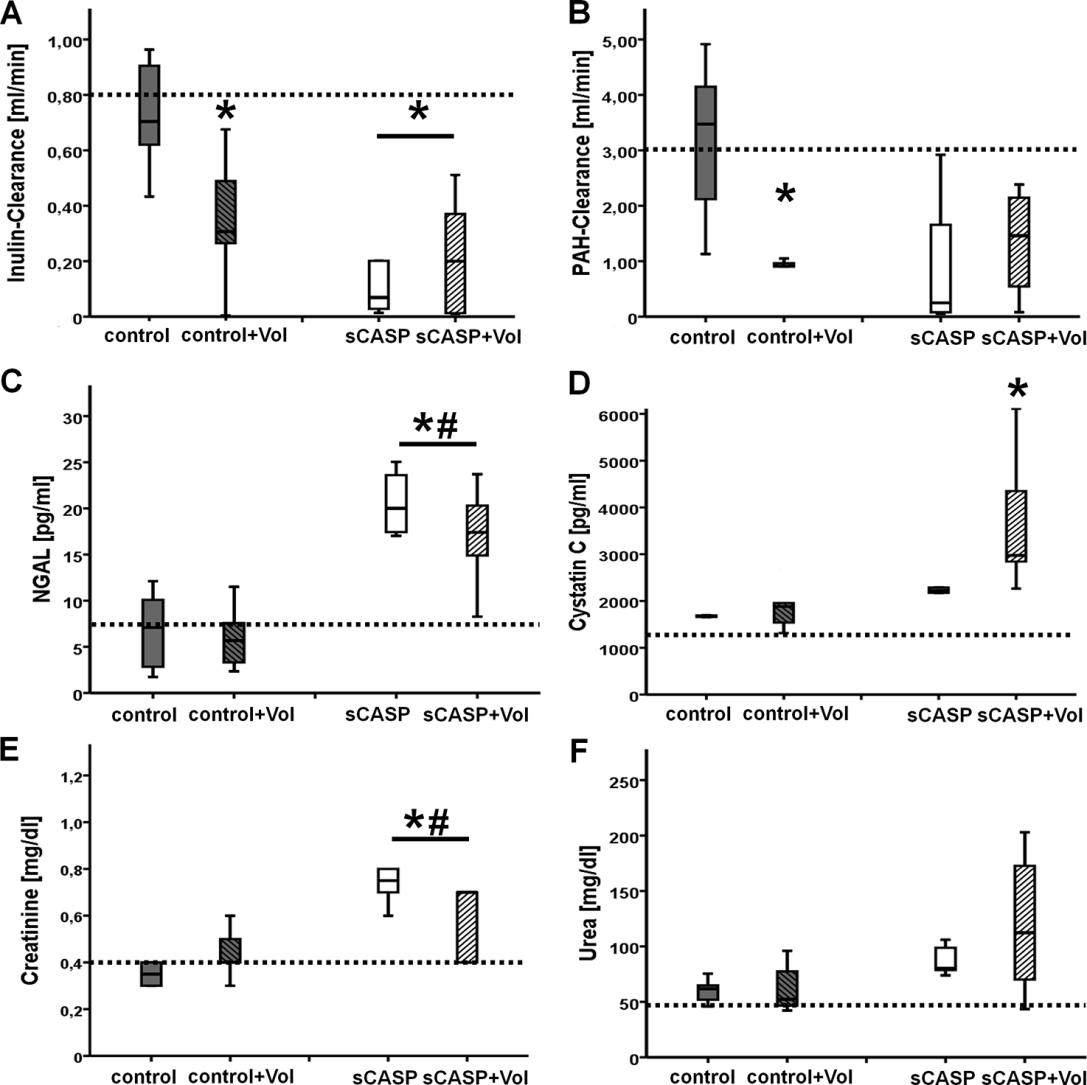
Evaluation of kidney function with serum parameters in vivo after 24 h. A: Inulin-Clearance [ml/min], the gold standard of kindey function; B: PAH-Clearance [ml/min]; C: NGAL [pg/ml]; D: Cystatin C [pg/ml]; E: Creatinine [mg/dl] F: Urea [mg/dl], n = 6, * p<0.05 vs. control, # p<0.05 vs. control+Vol. Dotted lines equal standard values of each parameter.

First we measured inulin-clearance as the gold standard of global kidney function (glomerular filtration rate). Administering 6% HES 130/0.4 to rats (C+Vol) resulted in a significantly reduced inulin-clearance compared to the control animals (0.35±0.25 vs. 0.72±0.19 [ml/min]) (Fig 3), while the inulin plasma concentrations showed no differences between the groups (169±28 vs. 171±18 [μl/l]). Additionally PAH-clearance was also significantly reduced to 0.84±0.26 [ml/min] in C+Vol when compared to control (3.16±1.53) (Fig 3). After 24h of sepsis the Inulin-Clearance of sCASP compared to sCASP+Vol showed no significant changes between each other as well as compared to control+Vol, but a significant decrease compared to control (0.20±0.29; 0.22±0.22; 0.35±0.25 vs. 0.72±0.19 [ml/min]) (Fig 3). No significant differences were detectable when comparing the inulin plasma concentrations (sCASP 239±79; sCASP+Vol 188±45; C+Vol 169±28; control 171±18 [μl/l]; p-value: 0.389). PAH-clearance did not reveal any significant variations (Fig 3), while PAH plasma concentrations showed also no significant differences: Control 78±28, C+Vol 43±7, sCASP 79±40 and sCASP+Vol 37±11 (μg/ml) (p-value: 0.059).

#### Urine output

While collecting the urine for the measurement of the Inulin- and PAH-Clearance, we also measured the amount of urine. Obviously C+Vol, sCASP and sCASP+Vol showed significantly decreased amounts of urine (0.11±0.03; 0.12±0.10; 0.12±0.13 [ml/20 min]) compared to control (0.36±0.14 [ml/20 min]). When related to body weight these significances were confirmed: 0.032±0.008, 0.041±0.038; 0.033±0.035 vs. 0.117±0.043 [ml/20 min/100g].

#### Serum markers of AKI

The weak standard parameters for AKI, creatinine [mg/dl] and urea [mg/dl] showed elevated levels, when comparing the two sCASP groups (sCASP, sCASP+Vol) to control and control+Vol ((88±14; 119±64 vs. 60±11; 63±23) (Fig 3); (0.77±0.14; 0.75±0.44 vs. 0.35±0.06; 0.44±0.11) (Fig 3)), whereas only creatinine reached significance. Measuring NGAL [pg/ml] revealed significantly increased levels at sCASP and sCASP+Vol animals compared to control and control+Vol animals ((21±4; 17±6 vs. 7±4; 6±4) (Fig 3)). Fianally cystatin C levels [pg/ml] showed no significant changes, apart from an increase comparing sCASP+Vol with control ((3721±1572 vs. 1676±105) (Fig 3)).

#### Histopathology

**Fig 4:**
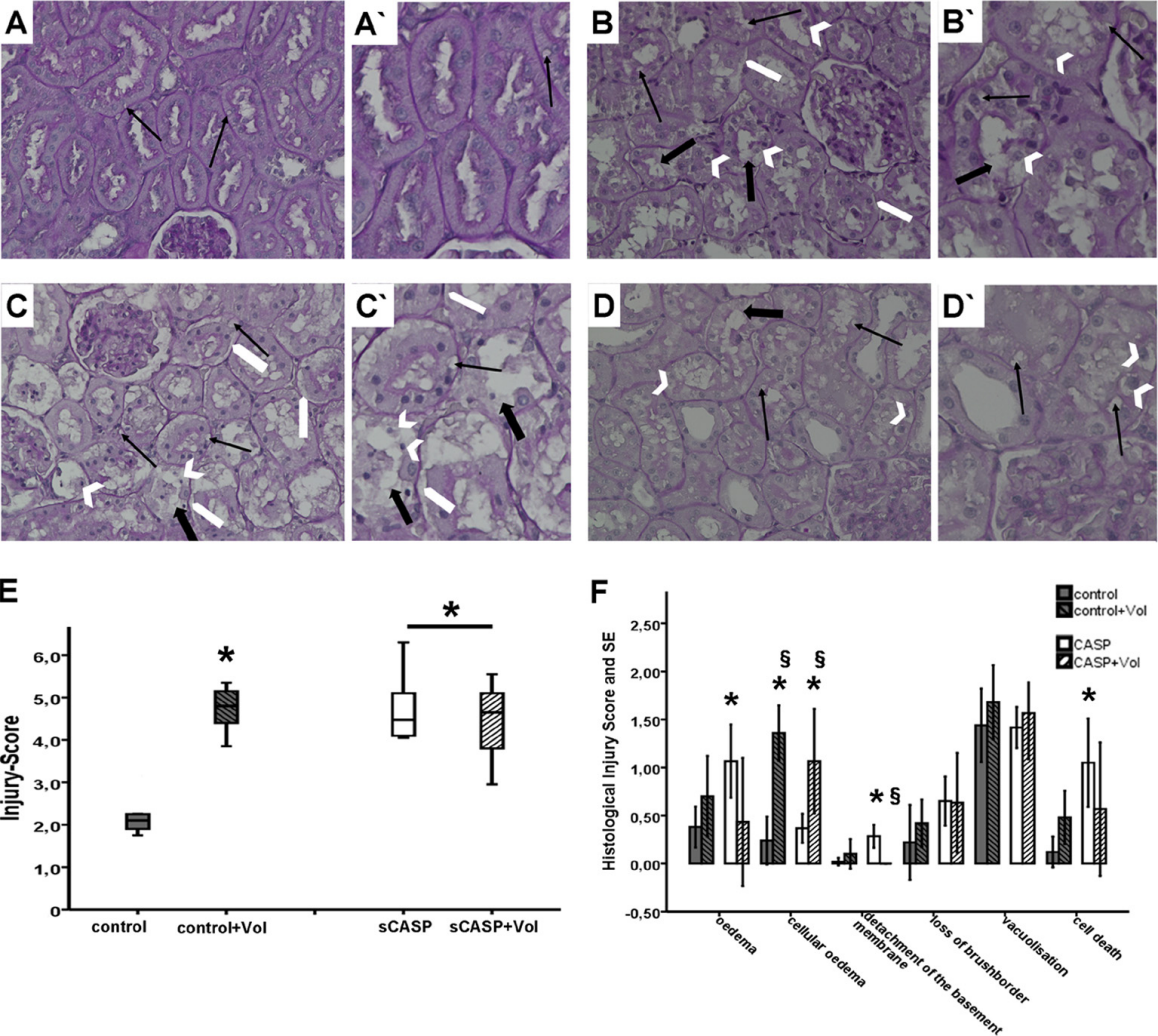
Representative images of the renal cortex following PAS reactions (A, B, C, D) and morphological alterations of the kidney after 24 h (E, F). A/A`: control, B/B`: control+Vol, C/C`: sCASP, D/D`: sCASP+Vol. Some of the lesion criteria are exemplified. Therefore, magnifications of some areas are shown (A`, B`, C`, D`). Black arrows showing vacuolization, white big arrows showing detachment of the basement membrane. White arrowheads showing cells going into cell death. Big black arrows detecting loss of the brush border of the proximal tubular cells. E: Total Injury Score: Criteria for histopathological assessment were formation of oedema, cellular oedema, detachment of tubular epithelium from the basement membrane, loss of the brush border of the proximal tubular cells, cell death and vacuolisation. Mean values from the scores of the latter criteria were taken together as total injury score. * p<0.05 vs. control. F: Single criteria of histopathological investigations. * p<0.05 vs. control, § p<0.05 vs. sCASP.

Infusion of 6% HES 130/0.4 in control animals (C+Vol) had a significant harmful impact on the kidney shown in the total injury score when compared to control (4.71±0.12 vs. 2.23±0.11) (Fig 4). Furthermore abdominal sepsis (sCASP, sCASP+Vol) increased histopathology significantly when compared to control (4.75±0.14; 4.38±0.44 vs. 2.23±0.11) (Fig 4), but there were no significant changes seen between the two groups compared to each other. In the kidneys of the C+Vol group significantly more cellular oedema and cell death is detectable compared to control. The kidneys of the sCASP group compared to the sCASP+Vol group, showed significantly more cases of detachment of the basement membrane of the proximale tubule cells as well as a tendency of more cell deaths and oedema. On the other hand, sCASP+Vol showed significantly more cellular oedema than sCASP.

## Discussion

The data of our survey, shows that HES impairs kidney function even under healthy conditions and inflammation seems not to be the key trigger for HES induced AKI in vivo. Fluid- and volume resuscitation remains a standard therapy in peri- and postoperative care. The choice of (invasive-) hemodynamic monitoring, type of solution and amount is an on-going, daily and controversial discussion[16, 17]. The theoretical advantage of colloid resuscitation with e.g. hydroxyethylstarch (HES) goes back to Starling’s equation in 1915[18]. In the last decades, numerous animal and human trials supported Starling’s theory. Because of these results, HES was the first choice for volume replacement in the OR as well as in the ICU in many countries for years. The VISEP-study started to show the other side of the story[1]. Further RCTs (6S, CRYSTMAS and CHEST-study) revealed increased risk for AKI using starch in septic patients [2–4], however these results were discussed controversially[19]. Parallel to the prohibition of HES in sepsis (EMA / 640658 / 2013), the validity of Starling´s theory began to falter[20]. It could be shown, that capillary colloid osmotic pressure gradient in the post-capillary venules (the theoretical force for fluid re-absorption into the vessels) is not as powerful as predicted by Starling’s equation[21]. Therefore the theoretical mechanisms about volume improvement with HES should be reconsidered.

Previously we demonstrated, that colloids (gelatine and HES) revealed histopathological changes mainly in the renal proximal tubule cells (PTC) in septic rats [8]. Because of reduced mortality and increased AKI parameters in these animals, we investigated the impact of HES in human PTCs in vitro. LPS- (Fig 1) as well as TNF-α [6] pretreatment had no influence on the HES induced reduction of PTC viability. Thus, these inflammatory settings seems not to be responsible for the harmful impact of colloids on PTC in vitro. Furthermore our results in vitro showed, that starch origin, molecular size, carrier solution or, older”HES-generation had marginal influence on the dose depended reduction of PTC viability in vitro [15]. Based on these results, the pure administrated mass of starch molecules seems to be the key problem for PTC in vitro. In line with that, a clinical investigation showed, that infusion of more than 33 ml/kgBW synthetical colloids lead to increased risk for AKI in septic patients [22]. Recently, it has been published, glomerular endothelium is damaged during inflammation which results in altered glomerular filtration[23]. Therefore HES might be more harmful in sepsis, due to the breakdown of the filter function in each glomerulum. As a result, more mass of HES molecules have faster contact to PTC leading to a more rapid incorporation from these kidney brushborder cells. Once HES is incorporated, PTC viability decreased immediately (data not shown) and cytotoxic HES effects increased over time (Fig 2). In theory, under healthy conditions, HES molecules < 70 kDa can pass the adult glomerulum and trigger a re-uptake by PTCs. However filtration barrier of the pediatric glomerulum is not completed and tubular function matures in the first year of life [24]. Accordingly starch molecules in the primary urine pass to PTCs and may lead to decreased viability in vivo. This hypothesis may be corroborated by the meta-analysis about the usage of HES in pediatric patients by Li et al. [25]. They concluded, that HES might have an adverse effect on renal function. However, in about 1000 children who received HES during cardiovascular surgery, no increase of AKI was detectable [26]. Witt et al. used 20 ml/kg HES in a pediatric piglet model and after 7 days, they did not find any relevant impact on renal function [27].

Therefore, we investigated in this study the impact of HES in sepsis compared to healthy conditions in our previously established novel animal setup of sCASP. Hereby we are able to screen macro haemodynamics, kidney function (inulin and PAH clearance) and kidney derangements (by serum parameters and histopathology) in the same animal [7]. Hypothetically, HES should be able to impair PTC function even under healthy conditions and sepsis should worsen this effect in vivo. In fact, 50 ml/kgBW 6% HES 130/0.4 over 6 h showed harmful side effects in healthy control rats. Interestingly, only inulin-, PAH-clearance, urine output as well as histopathology showed kidney injury. However, the routine parameters like urea, creatinine, NGAL or cystatin C remained normal. PAH clearance is used as an indirect measurement of renal plasma flow (RPF)[28]. PAH is glomerulary filtered and actively secreted by PTC. Thus we are not able to distinguish, whether HES reduced RPF or the reduction of PAH clearance is due to decreased PAH secretion by HES-induced viability reduction in PTC. Inulin and PAH plasma concentrations, the basis for clearance calculations, depend inter alia on the total blood volume. Since hematocrit (S2 Table) and total weight showed no differences between groups, HES treatment did not significantly influence total blood volume. Therefore individual volume status had no influence on the detected kidney function. Surprisingly, urine output decreased significantly in healthy rats by 50 ml/kg/BW HES. Therefore, we conclude, that HES can induce AKI in healthy rats by unknown mechanisms. As expected, HES reduced renal function in septic animals. Results from in vitro experiments and animal trials are not directly transferable into the human setting, but allow inspections, which are impossible to get from clinical human studies. Thus we postulate a diagnostic gap for HES induced AKI in clinical routine, because kidney biopsy in healthy adults for histopathology can/will never be performed in human HES trials.

There remain some possible limitations of this study. We demonstrated in our setup, that 6h of HES treatment reveals derangements of kidney function and morphology in control animals. However, with our setting, we cannot state a possible reversibility of HES induced AKI and the threshold of HES in sepsis, shock or other indications for colloids. Additionally our survey demonstrates that inflammation is no requisite for HES induced AKI. Thus, our hypothesis of a specific inflammatory driven signal as trigger of the pathomechanism of HES induced detrimental alterations in septic AKI are not verified.

## Conclusion

Despite of renal replacement therapy and improved intensive- and perioperative care medicine, development of AKI increases mortality and morbidity. There is growing evidence, that starch molecules induce AKI by unknown pathways. For the first time we demonstrated, that 50 ml/kgBW 6% HES 130/0.4 induced AKI within 6h in healthy rats. To proof the statement, that applying HES for volume resuscitation improves outcome, additional clinical trials and studies, focusing on adverse effects of HES regarding kidney function, are needed.

## Supporting Information

S1 Table Blood parameters at the end of the experiments. n = 6/group; *p<0.05 vs. control, # p<0.05 vs. control+Vol
(PDF)

S2 Table Arterial blood gas analysis (BGA): Baseline measurements were taken at time point 0 hours and after 24 h. N = 6/group, p<0.05 *vs. control, # vs. control+Vol, § vs. sCASP
(PDF)

S3 Table Macrohemodynamic measurements at 0 h (baseline) and after 24 h under general aneathesia and mechanical ventilation. HR = heart rate, MAP = invasive mean arterial blood pressure, BR = breathing rate, CI = cardiac index, SVI = stroke volume index, TPRI = total peripheral resistance index, DO_2_-I = oxygen delivery index. N = 6/group, *p<0.05 vs. control, # p<0.05 vs. control+Vol
(PDF)
