# Supplementary material for: Balanced Hydroxyethylstarch (HES 130/0.4) Impairs Kidney Function In-Vivo without Inflammation
Source: PLoS One. 2015 Sep 4;10(9):e0137247. doi: 10.1371/journal.pone.0137247 (PMC4560431; doi:10.1371/journal.pone.0137247)
Supplement: S1 Table — n = 6/group; *p<0.05 vs. control, # p<0.05 vs. control+Vol (PDF) [file pone.0137247.s001.pdf]

	sodium [mmol/L]	potassium [mmol/L]	calcium [mmol/L]	chloride [mmol/L]	osmolality [mosmol/kg]	glucose [mg/dl]	ASAT [U/L]	ALAT [U/L]	GGT [U/L]	Alkaline phosphatase [mmol/L]
**control**	142±3	5.3±0.7	2.53±0.05	108±5	298±5	111±19	82±34	34±5	2.1±2.5	92±12
**control+Vol**	139±4	4.7±0.6	2.44±0.15	104±4	292±7	144±26	168±244	41±15	2.4±2.5	103±22
**sCASP**	144±3#	5.0±0.6	2.32±0.21	111±3#	314±14#	141±41	534±454*	199±171*	6.8±7.6	160±98
**sCASP+Vol**	142±2	5.6±2.2	2.15±0.44	109±2#	313±11#	126±54	476±335*	114±79	4.1±3.4	112±44

	LDH [U/L]	protein [g/dl]	albumin [g/dl]	INR	PTT [sec.]	leukocyte [Thsd./µl]	erythrocyte [Mio./µl]	platelets [Thsd./µl]
**control**	169±90	4.0±0.2	2.3±0.2	0.79±0.01	23.1±10.6	3.28±1.15	5.78±0.41	255±89
**control+Vol**	321±471	4.2±0.6	2.3±0.3	0.78±0.06	19.9±2.1	2.48±0.71	6.06±0.87	281±128
**sCASP**	1075±1132	3.7±0.3	2.0±0.3*#	0.77±0.10	20.5±6.7	1.15±0.41	5.71±0.85	135±82
**sCASP+Vol**	894±556	3.8±0.6	1.9±0.3*#	0.83±0.10	20.3±1.3	1.26±0.48	5.98±1.22	183±193
