# Supplementary material for: Balanced Hydroxyethylstarch (HES 130/0.4) Impairs Kidney Function In-Vivo without Inflammation
Source: PLoS One. 2015 Sep 4;10(9):e0137247. doi: 10.1371/journal.pone.0137247 (PMC4560431; doi:10.1371/journal.pone.0137247)
Supplement: S2 Table — N = 6/group, p<0.05 *vs. control, # vs. control+Vol, § vs. sCASP (PDF) [file pone.0137247.s002.pdf]

BGA baseline	pH	pCO_2 [kPa]	pO_2 [kPa]	Hct [%]	Hb [g/dl]	sO_2 [%]	lactate [mmol/L]	HCO_3^- [mmol/L]	SBE [mmol/L]
**control**	7.45±0.02	5.73±0.47	12.19±0.38	46.7±2.0	15.3±0.7	96.0±0.4	1.1±0.2	29.1±1.5	5.1±1.3
**control+Vol**	7.45±0.04	5.01±0.71*	12.78±1.66	47.4±1.2	15.4±0.4	96.5±1.1	0.8±0.2	25.7±1.9*	2.1±1.6*
**sCASP**	7.38±0.02*#	6.50±0.57*#	13.05±0.89	51.3±4.7	16.9±1.5	95.1±1.3	1.8±0.5*#	27.7±2.1	3.2±1.7
**sCASP+Vol**	7.38±0.03*#	5.67±0.44#§	12.72±0.59	51.5±2.2	16.9±0.7	95.8±0.9	1.0±0.4§	24.5±1.2*	-0.1±1.3*#§

BGA 24 h	pH	pCO_2 [kPa]	pO_2 [kPa]	Hct [%]	Hb [g/dl]	sO_2 [%]	lactate [mmol/L]	HCO_3^- [mmol/L]	SBE [mmol/L]
**control**	7.44±0.02	5.18±0.50	24.51±2.99	38.8±3.4	12.6±1.1	97.5±0.3	1.6±0.2	26.0±1.2	2.2±0.9
**control+Vol**	7.45±0.06	4.81±0.84	19.86±4.84	36.9±4.3	12.0±1.5	97.1±1.0	1.8±0.5	24.5±1.8	0.9±1.6
**sCASP**	7.42±0.07	5.29±0.87	24.21±8.74	39.0±7.3	12.7±2.4	92.3±14.5	2.9±1.3	25.1±2.4	1.0±2.9
**sCASP+Vol**	7.28±0.20	6.52±2.41	16.06±5.26	39.4±1.8	12.8±0.6	93.1±8.1	3.0±4.1	21.4±4.3	-4.5±6.5
