# Supplementary material for: Balanced Hydroxyethylstarch (HES 130/0.4) Impairs Kidney Function In-Vivo without Inflammation
Source: PLoS One. 2015 Sep 4;10(9):e0137247. doi: 10.1371/journal.pone.0137247 (PMC4560431; doi:10.1371/journal.pone.0137247)
Supplement: S3 Table — HR = heart rate, MAP = invasive mean arterial blood pressure, BR = breathing rate, CI = cardiac index, SVI = stroke volume index, TPRI = total peripheral resistance index, DO2-I = oxygen delivery index. N = 6/group, *p<0.05 vs. control, # p<0.05 vs. control+Vol (PDF) [file pone.0137247.s003.pdf]

	HR[1] [beats/min]	MAP[1] [mmHg]	BR[1] [breaths/min]	HR [beats/min]	MAP [mmHg]	BR [breaths/min]	CI [ml/min/kg]	SVI [ml/beat/min]	TPRI [mmHg/ml/min/kg]	DO_2-I [ml/min/kg]
**control**	385±26	116±11	77±13	367±40	92±9	102±15	396±85	1.16±0.30	0.29±0.07	69.4±16.9
**control+Vol**	396±9	120±3	71±3	329±31	74±11	91±18	425±135	1.21±0.46	0.30±0.06	77.6±36.4
**sCASP**	384±42	128±7	64±10	457±49#	79±13	107±26	481±66	1.00±0.23	0.25±0.04	87.0±16.9
**sCASP+Vol**	380±31	125±9	65±8	367±71	67±24	104±16	563±179	1.44±0.63	0.18±0.07	93.8±26.0

1=baseline
